# A prognostic model for tumor recurrence and progression after meningioma surgery: preselection for further molecular work-up

**DOI:** 10.3389/fonc.2023.1279933

**Published:** 2023-11-01

**Authors:** Luis Padevit, Flavio Vasella, Jason Friedman, Valentino Mutschler, Freya Jenkins, Ulrike Held, Elisabeth Jane Rushing, Hans-Georg Wirsching, Michael Weller, Luca Regli, Marian Christoph Neidert

**Affiliations:** ^1^ Department of Neurosurgery, Clinical Neuroscience Center, University Hospital and University of Zurich, Zurich, Switzerland; ^2^ Department of Informatics, Eidgenössische Technische Hochschule (ETH) Zürich, Zurich, Switzerland; ^3^ Epidemiology, Biostatistics and Prevention Institute (EBPI), University of Zurich, Zurich, Switzerland; ^4^ Department of Neuropathology, University Hospital and University of Zurich, Zurich, Switzerland; ^5^ Department of Neurology, Clinical Neuroscience Center, University Hospital and University of Zurich, Zurich, Switzerland; ^6^ Department of Neurosurgery, Kantonsspital St. Gallen, St. Gallen, Switzerland

**Keywords:** meningioma, prediction model, immunohistochemistry, recurrence, progression, classification, preselection

## Abstract

**Purpose:**

The selection of patients for further therapy after meningioma surgery remains a challenge. Progress has been made in this setting in selecting patients that are more likely to have an aggressive disease course by using molecular tests such as gene panel sequencing and DNA methylation profiling. The aim of this study was to create a preselection tool warranting further molecular work-up.

**Methods:**

All patients undergoing surgery for resection or biopsy of a cranial meningioma from January 2013 until December 2018 at the University Hospital Zurich with available tumor histology were included. Various prospectively collected clinical, radiological, histological and immunohistochemical variables were analyzed and used to train a logistic regression model to predict tumor recurrence or progression. Regression coefficients were used to generate a scoring system grading every patient into low, intermediate, and high-risk group for tumor progression or recurrence.

**Results:**

Out of a total of 13 variables preselected for this study, previous meningioma surgery, Simpson grade, progesterone receptor staining as well as presence of necrosis and patternless growth on histopathological analysis of 378 patients were included into the final model. Discrimination showed an AUC of 0.81 (95% CI 0.73 – 0.88), the model was well-calibrated. Recurrence-free survival was significantly decreased in patients in intermediate and high-risk score groups (p-value < 0.001).

**Conclusion:**

The proposed prediction model showed good discrimination and calibration. This prediction model is based on easily obtainable information and can be used as an adjunct for patient selection for further molecular work-up in a tertiary hospital setting.

## Highlights:

- Recurrence prediction after meningioma surgery is feasible with few parameters.
- Our model identifies tumors requiring further molecular work-up.

## 1 Introduction

Meningiomas are the most common primary intracranial tumors and have a benign disease course in the greater part of cases (1). Most patients can be followed or in case surgery is needed, it is curative in the majority of cases without additional treatment (2). Primary treatment includes maximum safe resection, which may be followed by further therapy consisting usually of radiotherapy. Systemic therapies or even combinatorial approaches for high-grade tumors are not an established treatment options (2).

Conventional grading systems such as the WHO classification categorize patients into different grades of malignancy with increasing likelihood for tumor progression or recurrence (P/R) based mainly on histology. Molecular alterations as grading parameters have been introduced in 2021 (3, 4).

However, patient outcomes might still deviate from the prediction based on traditional classification systems leading to susceptible patients not being eligible for further treatment and vice-versa to patients with low recurrence risk potentially receiving additional therapy and putting them at an increased risk for unnecessary side effects.

Several slightly distinct integrated classification systems – mainly incorporating recently detected molecular profiling – have been described (5–8), all of which with a superior predictive ability compared to conventional scoring systems, based on histopathology. Nevertheless, molecular markers are on the uprise, however their daily clinical implementations have yet to be established and their assessment remains costly. Therefore clinical, histological, volumetric and immunohistochemical data remain the mainstay of easy-to-obtain and readily available meningioma classification data for the time being. By incorporating all this relevant patient information into a statistical prognostic model, a more accurate pre-selection of patients benefiting from further treatment compared to conventional classification systems might be achieved.

We here report the development of a prediction model for tumor P/R after microsurgical resection of meningiomas based on clinical, radiological, pathological and immunohistochemical data. The aim of this study was to provide a low-cost, easy to acquire and accurate prediction tool to select patient groups possibly benefitting from additional therapy after already having undergone microsurgical resection.

## 2 Materials and methods

### 2.1 Study design

We retrospectively analyzed prospectively collected data from our institutional patient registry of the Department of Neurosurgery at the University Hospital Zurich, a tertiary referral center (9). All patients aged 18 years or older undergoing surgery for the resection of an intracranial meningioma between January 2013 and December 2018 were screened for eligibility. Whenever the minimal 3-year (+/- 6 months) follow-up consultation had been completed, written patient consent was obtained and tumor samples were available for tissue microarray, patients were included. P/R was defined as recurrence of tumor after Simpson Grade I-III resections or increasing residual tumor size in Simpson grade IV – V on contrast enhanced T1-weighted MRI with a change in treatment strategy (10) within 3 years after initial surgery. Additional treatment was discussed in a weekly interdisciplinary tumor board and was applied according to the contemporary EANO guideline (11).

### 2.2 Study variables

All clinically relevant parameters available in the patient registry were extracted and contained age, sex, American Society of Anesthesiologists (ASA) risk classification, Karnofsky performance scale (KPS), modified Rankin scale (mRS), length of hospitalization, smoking status, complication at discharge and grading of the most severe complication according the Clavien-Dindo-Grade (CDG) (12). We extracted information on tumor calcification, edema, bone infiltration, cystic tumor parts, parenchymal infiltration and hyperostosis on CT and MRI scans from neuroradiological reports. Tumor and edema volume were measured using the SmartBrush application of Brainlab Elements, Brainlab AG Munich evaluated on contrast enhanced T1-weighted MRI as well as FLAIR/T2 sequences. The histological information was collected from the standardized neuropathological report containing WHO 2016 grade, mitotic index, MIB-1/Ki-67 index, nucleus-to-cytoplasmic (N/C) ratio and whether brain invasion, increased cellularity, nucleoli, patternless growth as well as necrosis were microscopically visible. Immunohistochemistry war performed as described in detail in Online resource 1. Simpson grade and Milan complexity score (13) were stratified after surgery in accordance with the main operating neurosurgeon. A prediction model for the binary outcome of P/R 3 years after surgery was built based on logistic regression analysis of all patients undergoing meningioma surgery from January 2013 to December 2018 as described in Online Resource 1. Logistic regression formula of the final model was used to create a scoring system, giving every predictor a score according to its regression coefficient and rounded to 0.5. Three risk categories were defined according to the predictive value. Scores between 0-20% probability were considered as low risk, 20-70% intermediate risk and >70% as high risk.

### 2.3 Immunohistochemistry

Formalin-fixed, paraffin-embedded (FFPE) tissue microarrays (TMA) composed of meningioma tumor samples were cut in 2 µm sections and stained using the BOND Fully Automated IHC Staining System or Ventana Roche BenchMark ULTRA Fully Automated IHC Staining System. Based on current literature and availability the sections were incubated with primary antibodies against Ki-67 (Cell Marque Lifescreen Ltd., Order no. 275R-16, dilution 1:100), p53 (DAKO A/S, order no. M7001, dilution 1:80), epithelial membrane antigen (EMA, DAKO A/S; order no. M0613, dilution 1:600), S-methyl-5’-thioadenosine phosphorylase (MTAP, Sigma-Aldrich; order no. WH0004507M1; dilution 1:100), somatostatin receptor (SSTR2, Zytomed Systems, order no. RBK046-05, dilution 1:25), progesterone receptor (Ventana-Roche, order no. 790-4296, prediluted dispenser), breast cancer 1-associated protein-1 tumor suppressor gene (BAP-1, Santa Cruz Biotechnology, Inc., order no. sc-28383, dilution 1:200), protein polybromo-1 (pBRM-1, Cell Signaling Technology, order no. 38439, dilution 1:100), phosphorylated histone H3 (pHH3, Abcam Limited, order no. ab32107, dilution 1:500), cd44 (PharMingen (Becton Dickinson), order no. 550392, dilution 1:100), Secreted frizzled-related protein 1 (SFRP-1, Abcam Limited, order no. ab4193, dilution 1:50), stathmin (Cell Signaling Technology, order no. #3352, dilution 1:50), neurofibromatosis-2 (NF-2, Sigma-Aldrich, order no. HPA003097, dilution 1:100) and Ras-related protein Rab-1B (RAB-1, St Johns Laboratory, order no. STJ140056, dilution 1:100). Visualization of the antibodies was performed with Bond Refine DAB Kit (Leica Bond) or Roche OptiView DAB detection kit. All sections were counterstained with hematoxylin. Staining extent was semiquantitatively scored as negative (0; <5% cells stained), focally positive (1; 5% to 50% cells stained) or diffusely positive mild intensity (2; >50%) under the supervision of an experienced neuropathologist (E.R.). The nuclear markers MIB-1/Ki-67 Index and pHH3 were quantified using Image J Software (ImageJ 1.52a, Wayne Rasband, National Institute of Health, USA, JAVA 1.8.0_172 (64-bit)) (14, 15).

### 2.4 Prediction model

A prediction model for the binary outcome of P/R 3 years after surgery was built based on logistic regression analysis of all patients undergoing meningioma surgery from January 2013 to December 2018. 390 patients met the inclusion criteria. Based on the current literature, clinical knowledge, and statistically significant values (16) in baseline analysis a preselection incorporating age, previous surgery, Simpson grade, skull base localization, radiological bone infiltration, MIB-1/Ki-67, mitotic index, histological increased NC ratio, present nucleoli, patternless growth, necrosis, EMA, and progesterone receptor stain collected pre- and postoperatively was put together, which was then considered for inclusion into the final model. Despite its statistically significant difference in univariate analysis mRS was not included due to its low availability in data collection. WHO grade itself was not considered due to its defining histopathological components being analyzed separately.

Some missing values in the candidate predictors were observed (3.2% in the variable MIB-1/Ki-67, 15.3% in EMA and 15.6% in progesterone staining). They were replaced with a non-parametric iterative imputation method using a random forest algorithm with the R package MissForest (17). In brief, the iterative imputation method commences with the value containing the smallest proportion of missing values. A random forest with the non-missing values of said value as dependent variable and all candidate predictors as independent variables is put together. Based on this, the missing values are imputed. Missingness at random was assumed.

Numerical variables were used as is for the prediction models, whereas for simplicity and clinical appliance, all categorical variables were dichotomized (yes/present and no/absent). MIB-1/Ki-67 was divided into a low (≤4%) and a high group (≥5%) as recently proposed in a systemic review incorporating more than 5000 patients (18). EMA and progesterone receptor stains were similarly categorized into low (negative and focally positive) and high staining (diffusely positive). Simpson grade was divided into gross total resection (grade I-III) and partial resection (grade IV and V). The Adaptive best subset selection for generalized linear model (abbess) package (19) was used for selection of the variables with the highest predictive ability. The number of predictive events was limited to 5 predictor variables according to the commonly used ten-to-one rule in relation of occurring events. K-fold cross-validation was used for internal validation. Overfitting was avoided by a variable preselection based on the current literature and statistically positive values in baseline analysis, by limiting the number of predictor variables to 5 and by k-fold cross-validation. Discrimination and calibration plots were created to assess the performance of the final model. Logistic regression formula of the final model was used to create a scoring system, giving every predictor a score according to its regression coefficient and rounded to 0.5. Prediction outcome can be calculated with the following formular $ŷ\;=\frac{exp\left(S\right)}{1+exp\left(S\right)}$ . For all possible scores from 1 to 6.5, the prediction score was calculated. Three risk categories were defined according to the predictive value. Scores between 0-20% probability were considered as low risk, 20-70% intermediate risk and >70% as high risk.

Reporting of the study is based on the Tripod Statement Checklist (20, 21) (Online Resource 1).

### 2.5 Statistical analysis

Continuous variables are given as means and standard deviation (SD) whereas categorical variables are reported as numbers and percentages of total, non-normally distributed data are depicted as median and inter-quartile range (IQR). All analyses were carried out using R version 4.2.1 (The R Foundation for Statistical Computing, Vienna, Austria) (22). A two-tailed p<0.05 was considered statistically significant. Chi-square-test, independent samples t-test and Mann-Whitney-U test were used for comparing patient groups. For Kaplan-Meier-curves progression/recurrence-free survival time (PFS) was noted for all patients, counted from date of surgery until last follow-up, the observation period lasted from 01.01.2013 until 31.12.2021. Patients without P/R still alive after observation ending were censored. All variables included into our final prediction model as well as all risk groups were compared for PFS.

### 2.6 Ethics

The scientific workup of patient data was approved upfront by the local ethics review board (Kantonale Ethikkommission Zürich, identifier PB-2017-00093) and is registered at https://clinicaltrials.gov (NCT01628406). Only patients with a written consent form were included. All procedures performed in studies involving human participants were in accordance with the Ethical Standards of the Institutional and/or National Research Committee and with the 1964 Helsinki Declaration and its later amendments or comparable ethical standards. This article does not report animal studies. All data are available on request.

## 3 Results

After applying our inclusion criteria 390 patients were included in our study. 342 patients had no P/R after surpassing 3-year clinical and radiological follow-up, whereas 48 patients showed P/R. The main baseline characteristics pre- and postoperatively are summarized in **Tables 1**, **2**. Patients with P/R were older at the date of surgery and had more frequently undergone previous meningioma surgery than patients with no P/R. Their functional status measured with the KPS and mRS tended to be lower. Radiological apparent bone infiltration was more frequent in P/R patient group, whereas skull base tumor location was more frequent in patients with no P/R.

**Table 1:** Baseline characteristics before surgery.

	Overall	No P/R	P/R	Exploratory p-value
n	390	342	48	
female patients, n (%)	292 (74.9)	261 (76.3)	31 (64.6)	0.115
Age, mean (SD)	57 (13.7)	57 (13.6)	62 (13.6)	0.011^*^
ASA, n (%)				0.268
1	48 (12.3)	46 (13.5)	2 (4.2)	
2	232 (59.5)	202 (59.1)	30 (62.5)	
3	108 (27.7)	92 (26.9)	16 (33.3)	
4	2 (0.5)	2 (0.6)	0 (0.0)	
KPS at admission (median [IQR])	90 [80.00, 90.00]	90 [80.00, 90.00]	90 [80.00, 90.00]	0.072^#^
mRS at admission (median [IQR])	1 [1.00, 2.00]	1 [1.00, 2.00]	1 [1.00, 2.00]	0.011^*^ **^,#^**
Previous neurooncological surgery (%)	61 (15.6)	35 (10.2)	26 (54.2)	<0.001^*^
Tumor diameter (median [IQR])	3.5 [2.40, 4.50]	3.5 [2.50, 4.50]	3.0 [2.35, 4.25]	0.308^#^
Radiological calcification visible (%)	61 (15.7)	58 (17.0)	3 (6.2)	0.088
Radiological bone infiltration visible (%)	50 (12.8)	39 (11.4)	11 (22.9)	0.045^*^
Radiological hyperostosis visible (%)	70 (17.9)	66 (19.3)	4 (8.3)	0.098
Radiological edema visible (%)	163 (45.8)	139 (44.6)	24 (54.5)	0.278
Radiological cystic parts visible (%)	24 (6.2)	22 (6.4)	2 (4.2)	0.771
Radiological parenchymal infiltration visible (%)	13 (3.3)	12 (3.5)	1 (2.1)	0.932
Tumor sidedness (%)				0.989
- left	182 (46.7)	160 (46.8)	22 (45.8)	
- middle	25 (6.4)	22 (6.4)	3 (6.2)	
- right	183 (46.9)	160 (46.8)	23 (47.9)	
Supratentorial location (%)	308 (79.0)	266 (77.8)	42 (87.5)	0.174
Tumor localisation (%)				
- skull base (%)	205 (52.6)	187 (54.7)	18 (37.5)	0.038^*^
- convexity	115 (29.5)	100 (29.2)	15 (31.2)	
- parasagittal	11 (2.8)	7 (2.0)	4 (8.3)	
- falx	38 (9.7)	32 (9.4)	6 (12.5)	
- tentorial	12 (3.1)	9 (2.6)	3 (6.2)	
- intraorbital	2 (0.5)	1 (0.3)	1 (2.1)	
- intraosseous	2 (0.5)	1 (0.3)	1 (2.1)	
- intraventricular	5 (1.3)	5 (1.5)	0 (0.0)	
Tumor volume preoperatively (cm^3^), (median [IQR])	12.67 [4.06, 33.03])	12.67 [4.22, 31.85]	12.35 [3.77, 37.75]	0.829^#^
Tumor edema preoperatively (cm^3^), (median [IQR])	0.00 [0.00, 17.07]	0.00 [0.00, 15.70]	1.62 [0.00, 26.97]	0.297^#^
Smoking status (%)				0.662
- current smoker	80 (22.7)	71 (23.3)	9 (18.8)	
- former smoker	51 (14.4)	45 (14.8)	6 (12.5)	
- non-smoker	222 (62.9)	189 (62.0)	33 (68.8)	
LOS (median [IQR])	7.00 [5.00, 10.00]	7.00 [5.00, 10.00]	7.00 [5.00, 9.00]	0.848^#^

All p-values <0.05 are marked with an asterisks (*) and non-normal distribution is marked with an ^#^. P/R, progression and/or recurrence; KPS, Karnofsky performance scale; mRS, modified Rankin Scale; LOS, length of hospital stay.

**Table 2:** Baseline characteristics after surgery.

	Overall	No P/R	P/R	Exploratory p-values
n	390	342	48	
Surgery duration (minutes, mean (SD))	273.91 (131.89)	273.63 (132.67)	275.88 (127.47)	0.912
Simpson grade (median [IQR])	2.00 [1.00, 3.00]	2.00 [1.00, 3.00]	2.00 [1.00, 4.00]	0.017^*,#^
Milan Complexity Score (median, [IQR])	3.00 [1.00, 4.00]	3.00 [1.00, 4.00]	3.00 [1.00, 4.00]	0.830^#^
Histology (%)				<0.001^*^
- anaplastic	9 (2.3)	4 (1.2)	5 (10.6)	
- angiomatous	3 (0.8)	3 (0.9)	0 (0.0)	
- atypical	80 (20.6)	63 (18.4)	17 (36.2)	
- chordoid	4 (1.0)	4 (1.2)	0 (0.0)	
- clear cell	2 (0.5)	1 (0.3)	1 (2.1)	
- fibrous or fibroblastic	27 (6.9)	25 (7.3)	2 (4.3)	
- meningothelial	129 (33.2)	118 (34.5)	11 (23.4)	
- microcystic	6 (1.5)	6 (1.8)	0 (0.0)	
- not otherwise specified	18 (4.6)	18 (5.3)	0 (0.0)	
- psammomatous	5 (1.3)	5 (1.5)	0 (0.0)	
- rhabdoid	2 (0.5)	1 (0.3)	1 (2.1)	
- secretory	8 (2.1)	8 (2.3)	0 (0.0)	
- transitional or mixed	96 (24.7)	86 (25.1)	10 (21.3)	
MIB-1/Ki-67 rate (median [IQR])	6.00 [3.00, 10.00]	5.00 [3.00, 10.00]	10.00 [5.00, 29.75]	<0.001^*,#^
Mitoses visible on histology (median [IQR])	1.00 [0.00, 3.00]	1.00 [0.00, 3.00]	3.00 [1.00, 7.00]	<0.001^*,#^
Brain invasion visible on histology (%)	33 (8.5)	24 (7.1)	9 (19.1)	0.012^*^
Increased cellularity visible on histology (%)	79 (20.4)	69 (20.3)	10 (21.3)	1
Nuclear-cytoplasmic ratio visible on histology (%)	20 (5.2)	14 (4.1)	6 (12.8)	0.031^*^
Nucleoli visible on histology (%)	99 (25.6)	77 (22.6)	22 (46.8)	0.001^*^
Patternless growth visible on histology (%)	22 (5.7)	13 (3.8)	9 (19.1)	<0.001^*^
Necrosis visible on histology (%)	62 (16.0)	43 (12.6)	19 (40.4)	<0.001^*^
EMA stain (%)				0.001^*^
- 0	43 (13.1)	31 (10.8)	12 (30.0)	
- 1	84 (25.7)	72 (25.1)	12 (30.0)	
- 2	200 (61.2)	184 (64.1)	16 (40.0)	
MTAP stain (%)				0.464
- 0	10 (3.1)	8 (2.8)	2 (4.9)	
- 1	47 (14.6)	39 (13.8)	8 (19.5)	
- 2	266 (82.4)	235 (83.3)	31 (75.6)	
sstr2A stain (%)				0.396
- 0	34 (10.7)	32 (11.4)	2 (5.3)	
- 1	34 (10.7)	31 (11.0)	3 (7.9)	
- 2	251 (78.7)	218 (77.6)	33 (86.8)	
Progesterone receptor stain (%)				0.003^*^
- 0	71 (21.8)	61 (21.3)	10 (25.0)	
- 1	56 (17.2)	42 (14.7)	14 (35.0)	
- 2	199 (61.0)	183 (64.0)	16 (40.0)	
BAP-1 stain (%)				0.1
- 0	9 (2.8)	9 (3.1)	0 (0.0)	
- 1	34 (10.4)	33 (11.5)	1 (2.5)	
- 2	283 (86.8)	244 (85.3)	39 (97.5)	
pBRM-1 stain (%)				0.374
- 0	37 (11.5)	31 (11.0)	6 (15.0)	
- 1	54 (16.7)	45 (15.9)	9 (22.5)	
- 2	232 (71.8)	207 (73.1)	25 (62.5)	
p53 (median [IQR])	3.00 [1.00, 6.00]	3.00 [1.00, 6.00]	4.00 [2.00, 7.00]	0.107^#^
pHH3 (median [IQR])	3.00 [1.00, 8.00]	3.00 [1.00, 8.00]	2.00 [1.00, 6.25]	0.118^#^
cd44 stain (%)				0.275
- 0	191 (58.4)	163 (56.8)	28 (70.0)	
- 1	97 (29.7)	88 (30.7)	9 (22.5)	
- 2	39 (11.9)	36 (12.5)	3 (7.5)	
SFRP-1 staining intensity 1 vs 0 (%)	16 (4.9)	15 (5.2)	1 (2.6)	0.744
STMN-1 stain (%)				0.939
- 0	47 (14.6)	41 (14.4)	6 (15.4)	
- 1	141 (43.7)	125 (44.0)	16 (41.0)	
- 2	135 (41.8)	118 (41.5)	17 (43.6)	
NF-2/Merlin stain (%)				0.585
- 0	21 (6.5)	20 (7.0)	1 (2.6)	
- 1	66 (20.5)	58 (20.4)	8 (21.1)	
- 2	235 (73.0)	206 (72.5)	29 (76.3)	
KPS at discharge (median [IQR])	90 [80.00, 90.00]	90 [80.00, 90.00]	90 [80.00, 90.00]	0.080^#^
mRS at discharge (median [IQR])	1.00 [0.00, 2.00]	1.00 [0.00, 2.00]	1.00 [1.00, 2.00]	0.049^*,#^
Complication at discharge (%)	115 (29.5)	100 (29.2)	15 (31.2)	0.907
Worst CDG at discharge (mdian [IQR])	1.00 [1.00, 2.00]	1.00 [1.00, 2.00]	1.00 [1.00, 2.00]	0.783^#^

All p-values <0.05 are marked in asterisks (*) and non-normal distribution is marked with an #. CDG, Clavien-Dindo-Grade.

Postoperatively, Simpson grade was higher in the patient group with P/R. The same is true for WHO grade, MIB-1/Ki-67, mitosis rate, histological brain invasion, N/C ratio, number of nucleoli, patternless growth and necrosis. Intense EMA stain and progesterone receptor stain were less frequent in the P/R patient group.

All patient data sets were used for baseline characteristics analysis. According with the literature and preliminary analysis age, previous surgery, MIB-1/Ki-67, mitoses rate, N/C ratio, the presence of nucleoli, patternless growth, necrosis and radiological bone infiltration, EMA and progesterone receptor stain, Simpson grade as well as skull base localization served as a preselection warranting consideration for inclusion into the final prediction model. Multiple imputation by a random forest algorithm was used whenever non-available values were encountered. Due to a high mean squared error in imputed mitoses values and lower model performance in regards of discrimination and calibration, all 12 non available mitoses data points were not considered for analysis resulting in 378 complete patient sets for prediction modelling. Proportion of falsely classified entries (PFC) for imputed MIB-1/Ki-67 resulted at 0.38, for EMA at 0.39 and progesterone receptor at 0.37. The abess package in R was used with cross validation to rank variables according to their predictive ability. The top 5 variables previous surgery, presence of microscopically necrosis, and patternless growth as well as progesterone receptor stain and Simpson grade were further incorporated into our final logistic regression model. The y-intercept of the final model was at -2.69, logistic coefficients were 1.26 for patternless growth, 1.1 for necrosis, -0.87 for progesterone receptor staining, 0.81 for Simpson grade and 2.15 for previous neurooncological surgery. After rounding all coefficients to 0.5 the risk score function was obtained, and binary predictor values could be filled in resulting in a P/R predictive score ranging from 0 to 1, which was further divided into 3 risk categories. Outcome prediction can be calculated with the following formula $ŷ\;=\frac{exp\left(S\right)}{1+exp\left(S\right)}$ , S = -2.69 + 1.5 x patternless growth (yes/no) + 1 x necrosis (yes/no) – 1 x progesterone receptor stain (low/high) + 1 x Simpson grade (total resection (I-III)/partial resection (IV/V)) + 2 x previous surgery (yes/no).

Use of the prediction model

All individual points of scoring systems are depicted in **Table 3** alongside their respective prediction scores and risk groups.

**Table 3:** Scoring points with predicted chance of P/R and their respective risk group.

Score	Predicted P/R probability	Risk group	Number of Patients
0	6%	1	133
1	16%	2	100
1.5	23%	2	6
2	33%	2	49
2.5	45%	2	4
3	58%	2	14
3.5	69%	2	4
4	79%	3	9
4.5	86%	3	3
5	91%	3	1
5.5	94%	3	2
6	96%	3	0
6.5	98%	3	1

**Figure 1:**
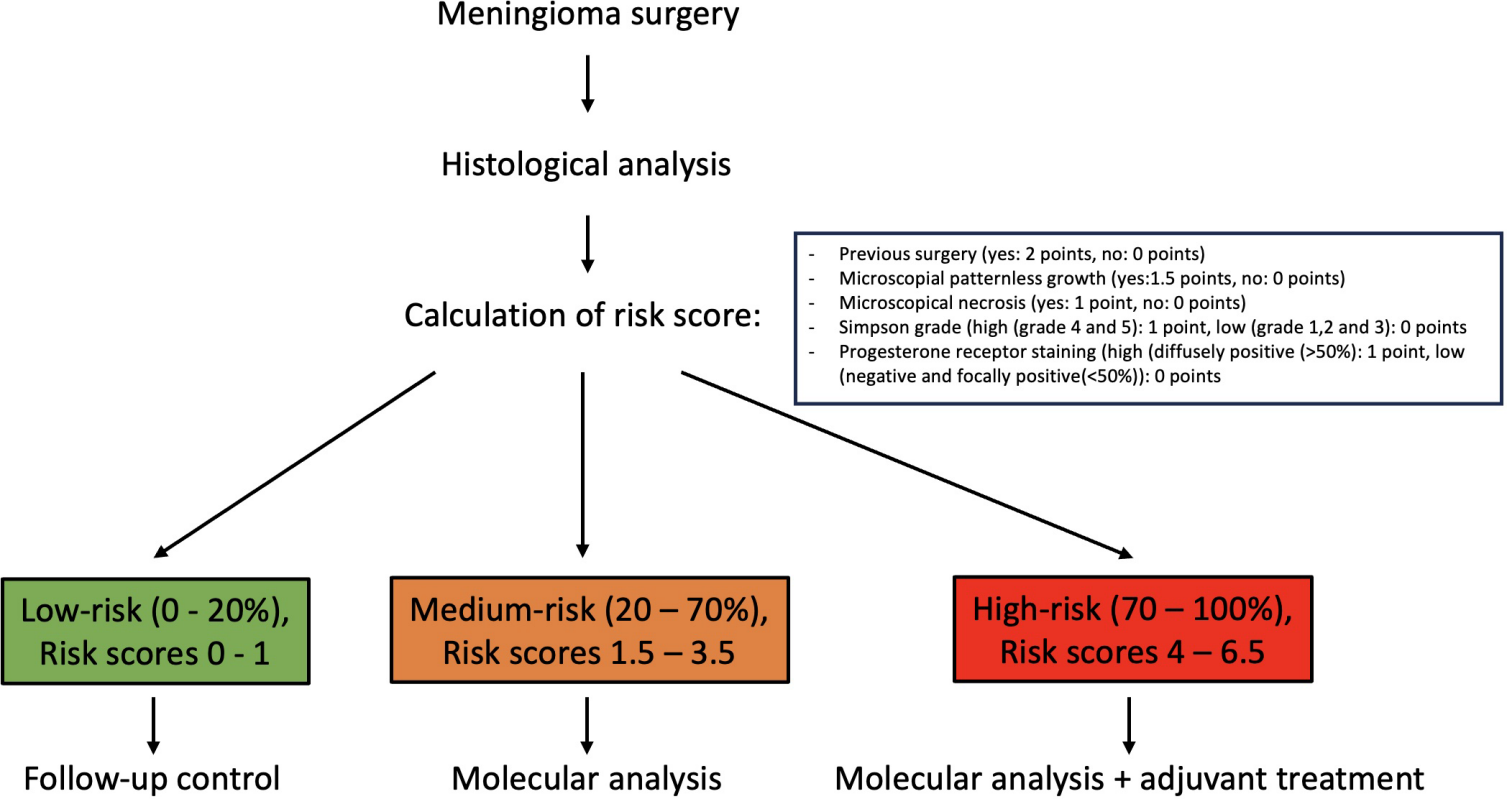
Flowchart and clinical use of proposed risk score.

A calculation example is provided in the following. A 64-year-old female patient presents with a one-time epileptic seizure without neurological history. On imaging a left frontal extra-axial tumor mass is detected and removed without complication in a Simpson grade I resection. Histological workup reveals patternless growth and necrotic spots, yet no other atypical features are present which is why the tumor is graded as WHO grade I and no further therapy is recommended. The patient’s recurrence risk is at S= -2.69 + 1.5 (patternless growth) + 1 (necrosis) = -0.19 which results in a predicted P/R probability of 45%. Further molecular work-up should be recommended in this patient example. A flowchart of clinical use of the proposed scoring system is depicted in **Figure 1**.

**Figure 2:**
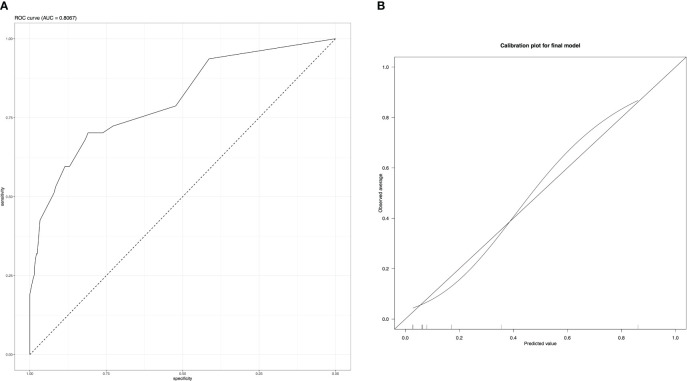
ROC analysis **(A)** and calibration curve **(B)** of the final model.

Area under the curve (AUC) of the final model (**Figure 2A**) was 0.81 (CI 0.73 – 0.88), with a threshold of 0.2 on receiver operation characteristic (ROC) analysis, sensitivity was 0.53 and specificity 0.92. Increasing the threshold to 0.5 a sensitivity of 0.28 with a specificity of 0.98 was reached with 0.7 threshold the sensitivity dropped to 0.15 with a slightly increased specificity of 1. The calibration plot showed a good prediction of the final model (**Figure 2B**). Brier score was at 0.08.

**Figure 3:**
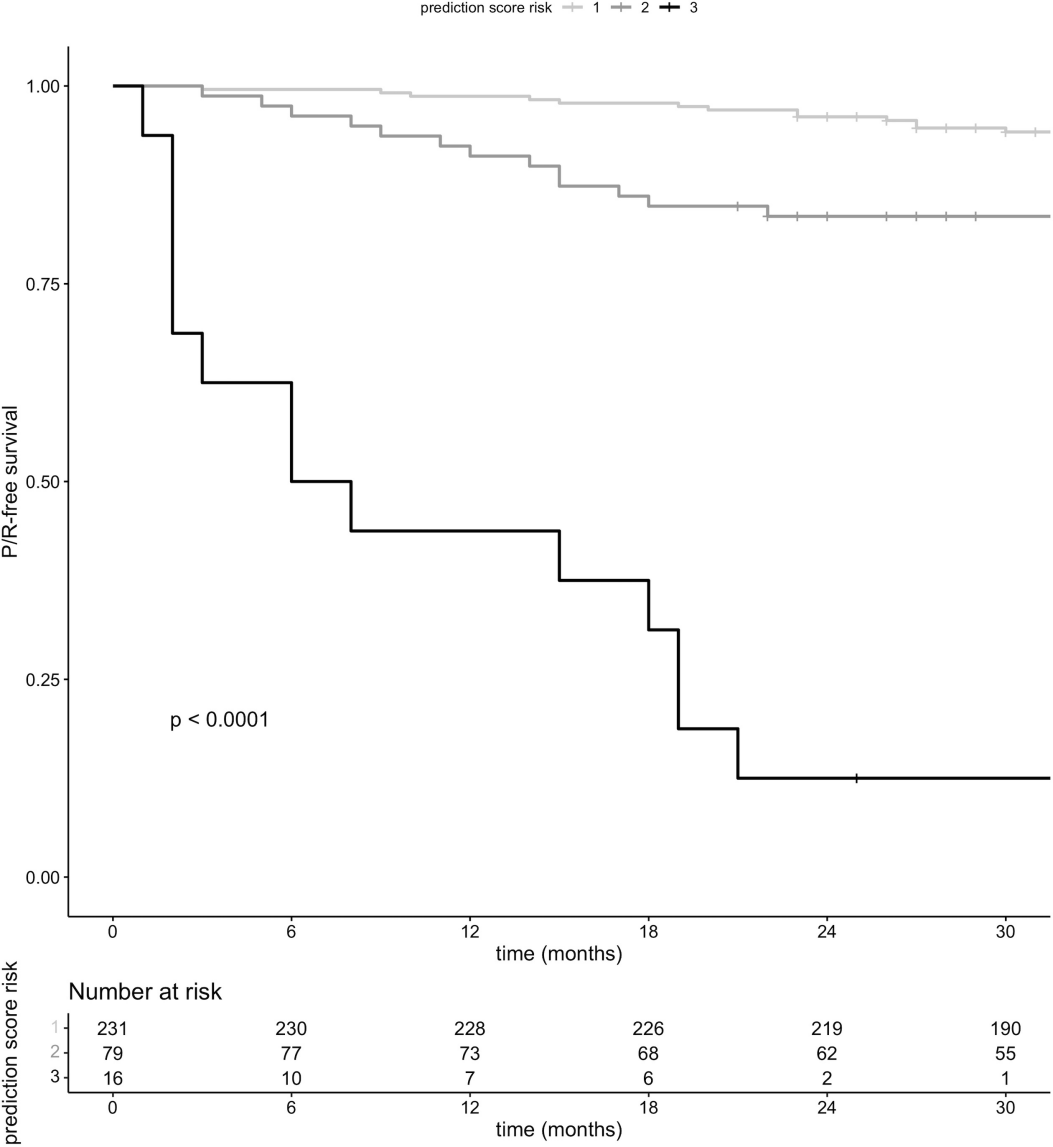
P/R-free survival stratified by risk groups.

P/R-free survival was significantly lower in patients who fell into risk groups two and three, respectively, compared to risk group one (**Figure 3**).

**Figure 4:**
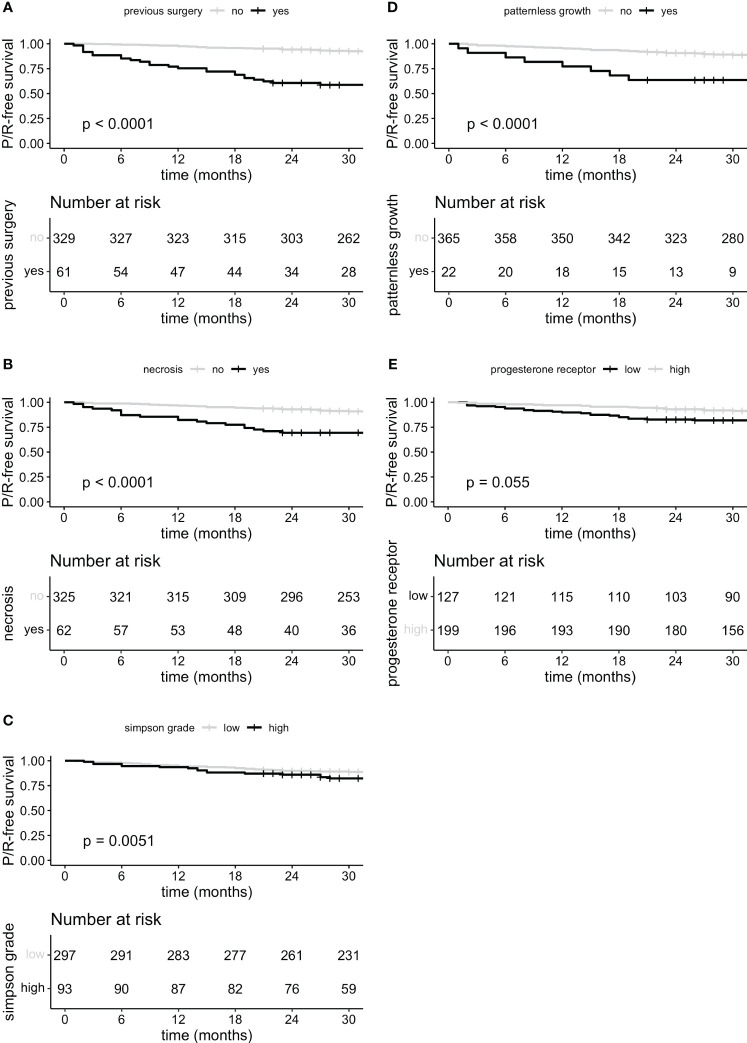
P/R-free survival curves for predictor variables previous surgery **(A)**, microscopically necrosis **(B)**, Simpson grade grouped **(C)** low (I, II and III) and high (IV and V), microscopically patternless growth **(D)** and progesterone receptor staining **(E)** stratified in low staining expression (negative and focally positive <50%) and high expression (>50%).

Furthermore, P/R-free survival was significantly lower in patients with previous surgery, necrosis, patternless growth on histopathological analysis as well as higher Simpson grade, progesterone receptor staining remained without significant difference in survival (**Figures 4A–E**).

Comparing the absolute number of each WHO grade in risk groups, 16% of all low-risk patients consisted of WHO grade II tumors, whereas patients at medium-risk were composed of 54% of WHO grade I tumors (**Table 4**). Kaplan-Meier-curve was similar for WHO classification and risk groups respectively.

**Table 4:** WHO grades stratified into the different risk groups.

	Low-risk group	Medium-risk group	High-risk group
WHO grade 1	193 (81%)	43 (18%)	3 (1%)
WHO grade 2	38 (49%)	31 (40%)	9 (11%)
WHO grade 3	0 (0%)	5 (56%)	4 (44%)

## 4 Discussion

Meningiomas have a good prognosis with low recurrence and/or progression rates. It is therefore crucial to properly identify susceptible patients for P/R who are likely to benefit from additional treatment. Traditionally, risk stratification for meningiomas has primarily been based on histopathological criteria, the most recent 2021 WHO Classification of Tumors of the Central Nervous System has additionally endorsed molecular markers, following the general trend in neuro-oncological classification. Other than *TERT* promoter mutation (4) and homozygous *CDKN2A/B loss (* 23), both leading to a WHO grade 3, the relevance of genetic alterations is yet to be fully established.

In recent years, a number of studies were aimed at the development of novel, more accurate prediction models of tumor biology and disease course by incorporating variables beyond histopathological criteria. DNA methylation profiling as well as copy-number variations (CNV) have been shown to predict progression and prognosis in meningioma effectively and more precisely than WHO grades (5–8, 24–27). A recently proposed new classification system has incorporated histological, molecular, methylation markers as well as CNV (6). Its reported prediction accuracy was superior to histopathological classification systems.

Nassiri et al. (28) developed a new classification system focusing mainly on molecular cluster groups with DNA somatic copy-number aberrations, DNA somatic point mutations, methylation, and mRNA abundance. A more accurate PFS prediction score was reached with this approach. High-quality clinical data have also been accounted for in this study. In contrast, Bayley et al. (29) focused on RNA-sequencing and cytogenetics which similarly lead to more accurate in prediction of tumor biology compared to conventional histopathology. Furthermore, comparing these methods to DNA methylation they concluded that each of the techniques identified similar patient groups, two benign and one malignant one. Similarly, Patel et al. (30) applied an unsupervised approach to RNA sequencing which revealed 3 cluster types all with better predictive ability of PFS than WHO classification. Importantly, both this model and the one described by Sahm et al. (26) do not rely on the WHO classification. Despite the emergence of novel meningioma classification systems – mainly incorporating molecular data – a consensus for general applicability has yet to be reached. However, a paradigm shifts from histological to molecular meningioma diagnostics is likely to only be a matter of time.

Even though molecular profiling and DNA methylation (7, 26) seems to be the future gold standard for meningioma diagnostics, their implementation into daily clinical practice has not yet been established. The costs associated with these novel forms of classification might increase healthcare costs, and its implementation in developing countries may prove even more challenging. Once implemented, it remains questionable whether all treated patients need to undergo molecular profiling due to the high rate of benign courses of surgically treated meningioma cases. Careful selection of patients to identify those at risk for P/R where molecular profiling and improved recurrence risk prediction might be warranted. Here, we introduced a simple-to-use and cost-effective binomial logistic regression model with a corresponding risk score with a high predictive ability. It relies on few and easily obtainable predictor variables, making its implementation in daily practice viable. By additionally proposing a scoring system in a simple three-class fashion, patients with intermediate risk scores might warrant for further molecular diagnostics, whereas high risk patients might go for additional therapy directly. The fact that in our cohort some WHO grade I tumors were categorized as medium or even high-risk and WHO grade II tumors as low-risk while maintaining similar survival curves on Kaplan-Meier-analysis undermines the added value of our proposed prediction model and risk score.

The predictive ability of molecular alterations in meningioma classification is striking. It seems therefore crucial to include molecular profiles in some form into any P/R prediction tool. Surrogate immunohistochemical markers in meningioma diagnostics have been described (31, 32) and might be a potential low-cost alternative for direct molecular profiling. Several molecular mutations have been integrated for the first time in 2021 WHO meningioma classification. Broadly, they can be dichotomized as NF2 mutation and non-NF2-mutated meningioma due to the high prevalence of NF2 mutations. Among the most common non-NF2 mutations are TRAF7, KLF4, AKT1, PIK3CA, POLR2A, SMO, CDKN2A/CDKN2B and TERT promoter mutations (33–35). Other less frequent ones have been described. AKT1 and KLF are often associated with TRAF7 mutations and most commonly occur in WHO grade 1 tumors.

Identifying known molecular alterations or their transcripts with immunohistochemical antibodies are a low-cost and feasible alternative to whole genome sequencing. SFRP-1 for instance, has been shown to be upregulated in AKT1(E17K) activating mutation which predominantly appears in WHO 1 meningiomas (32). Stathmin-1 expression on the other hand is a known marker of PI3K-AKT pathway activation and is increased in AKT1 mutated meningiomas (31) also showing tendency towards beneficial prognosis. MTAP immunohistochemistry has been described as a surrogate marker for homozygous CDKN2A loss correlating with higher graded meningiomas (36). Biallelic inactivation of PRBM 1 has been proposed to have a higher occurrence in WHO grade 3 papillary meningiomas (37), anti-PRBM1 antibody therefore possibly being a surrogate marker for poor prognosis in terms of recurrence. BAP1 mutations are linked with rhabdoid meningiomas therefore its antibody might be associated with poor prognosis (38, 39). CD44 has been reported to be expressed more frequently in higher graded meningiomas (40, 41). SSTR2 and EMA are commonly used to histologically differentiate meningiomas from other neurooncological tumors. However, due to their high frequency of expression in meningioma, their predictive power is not as high as other immunohistochemical stains. Progesterone receptors are expressed more frequently in lower graded tumors (42–44). The same was also true for our patient cohort and the effect was as great as that it showed to be an accurate predictor of P/R. Apart from EMA and progesterone receptor staining all remaining immunohistochemical markers did not show any difference in baseline characteristics between patient groups.

The final binomial logistic prediction model revealed five readily available predictor variables with high predictive ability and good discrimination. As mentioned above, the literature on progesterone receptor points toward a less frequent expression of progesterone receptors in higher-graded meningiomas. Simpson grade remains an important independent predictor of PFS (45, 46). Similarly, histological evidence of necrosis and patternless growth or so-called sheeting have already been described to increase risk of recurrence. Both variables have been included in meningioma risk scores before (47, 48). Literature on predictive ability for patients having undergone previous meningioma resection surgery is scarce. However, estimating the risk of P/R is crucial and adequate preselection of susceptible patients for further therapy is warranted. Even though most of our proposed predictor variables have been described before, further and prospective validation studies are needed to confirm the results before clinical implementation.

We are aware of some notable limitations of our research. Even though all data were collected prospectively, the final analysis was designed as a retrospective study, therefore all limitations for this study design are also valid for our analysis. Furthermore, available data especially number of total events for analysis was limited with a big difference to the number of non-events and was only collected for one tertiary referral hospital. This is why external validation and bigger cohort studies are necessary to further increase the validity of our prediction analysis.

All missing values were multiply imputed via random forest with is the gold standard for imputation yet still holds the risk of bias. The risk score was computed based on logistic regression coefficients. For simplicity all coefficients were rounded to 0.5 which also may over- or underestimate the importance of each predictor variable. Regarding data collection, it has to be noted that even though grading of immunohistochemical staining was based on objective predefined criteria, it remains an observer-specific measure. To minimize risk of bias all analysis were carried out in accordance and under supervision of an experienced neuropathologist.

Overall, we believe that our model, which uses easy to gain and low-cost variables could be used as a pre-selection guide for further molecular diagnostics and thus will support clinical decision making.

## 5 Conclusions

The predictive ability of genetic alterations in meningioma is deemed high, but their integration into the WHO classification remains to be established. Molecular assays however are not readily available in all centers and might further increase treatment costs. Predictive tools might provide a cost-effective alternative for pre-selection of patients at higher recurrence risk benefitting from treatment beyond surgery. By considering clinical, radiological, and histological as well as immunohistochemical parameters an accurate, well calibrated prediction model with good discrimination was created based on the variables: previous surgery, Simpson grade, progesterone receptor staining as well as presence of necrosis and patternless growth. These easy-to-obtain and cost-friendly variables potentially may guide selection for further molecular diagnostics and support clinical decision making.

## Data availability statement

The raw data supporting the conclusions of this article will be made available by the authors, without undue reservation.

## Ethics statement

The studies involving humans were approved by Kantonale Ethikkommission Zürich. The studies were conducted in accordance with the local legislation and institutional requirements. The participants provided their written informed consent to participate in this study.

## Author contributions

LP: Writing – original draft, Writing – review & editing, Conceptualization, Data curation, Formal Analysis, Investigation, Methodology. FV: Supervision, Writing – original draft, Writing – review & editing. JF: Conceptualization, Data Curation, Formal Analysis, Writing – review & editing. VM: Writing – review & editing, Data curation. FJ: Writing – review & editing, Data curation. UH: Writing – review & editing, Conceptualization, Formal Analysis. ER: Writing – review & editing, Data curation, Formal Analysis. H-GW: Writing – review & editing, Data curation. MW: Writing – review & editing. LR: Writing – review & editing. MN: Conceptualization, Methodology, Supervision, Writing – original draft, Writing – review & editing.
